# 6-Gingerol induces cell-cycle G1-phase arrest through AKT–GSK 3β–cyclin D1 pathway in renal-cell carcinoma

**DOI:** 10.1007/s00280-019-03999-9

**Published:** 2019-12-12

**Authors:** Shan Xu, Haibao Zhang, Tianjie Liu, Wenjie Yang, Wei Lv, Dalin He, Peng Guo, Lei Li

**Affiliations:** 1 Department of Urology, The First Affiliated Hospital of Xi’an Jiaotong University, 277 Western Yanta Road, Xi’an, 710061 Shaanxi People’s Republic of China; 2 Oncology Research Lab, Key Laboratory of Environment and Genes Related to Diseases, Ministry of Education, Xi’an, People’s Republic of China; 3 Key Laboratory for Tumor Precision Medicine of Shaanxi Province, Xi’an Jiaotong University, Xi’an, People’s Republic of China

**Keywords:** RCC, 6-gingerol, G1 arrest, AKT, GSK 3β

## Abstract

**Purpose:**

6-Gingerol, a major biochemical and pharmacological active ingredient of ginger, has shown anti-inflammatory and antitumor activities against various cancers. Searching for natural products with fewer side effects for developing adjunctive therapeutic options is necessary.

**Methods:**

The effects of 6-gingerol on proliferation, colony formation, and cell cycle in RCC cells were detected by a 3-(4,5-dimethylthiazol-2-yl)-2,5-diphenyltetrazolium bromide (MTT) assay, colony formation assay, and propidium iodide (PI) staining, respectively. Western blotting, an immunofluorescence assay, and immunohistochemical staining were performed to assess the expression of relevant proteins. A subcutaneous tumor model was set up to investigate the 6-gingerol effects on tumor growth in vivo, and the pharmacokinetics of 6-gingerol in mice were detected by LC/MS assays.

**Results:**

6-Gingerol treatment exerted time- and dose-dependent inhibition of the growth and colony formation of ACHN, 786-O, and 769-P cells, leading to a concomitant induction of cell-cycle G1-phase arrest and decrease in Ki-67 expression in the cell nucleus. Western-blotting results showed that 6-gingerol reduces phosphorylation of protein kinase B (AKT) Ser 473, cyclin-dependent kinases (CDK4), and cyclin D1 and, meanwhile, increases glycogen synthase kinase (GSK 3β) protein amount. Furthermore, the efficacy of 6-gingerol was demonstrated in an in vivo murine model of 786-O.

**Conclusion:**

The above results indicate that 6-gingerol can induce cell-cycle arrest and cell-growth inhibition through the AKT–GSK 3β–cyclin D1 signaling pathway in vitro and in vivo, suggesting that 6-gingerol should be useful for renal-cell carcinoma treatment.

**Electronic supplementary material:**

The online version of this article (10.1007/s00280-019-03999-9) contains supplementary material, which is available to authorized users.

## Introduction

The incidence of renal-cell carcinoma (RCC) has increased rapidly among both men and women according to the American Cancer Society. In general, patients with RCC benefit from conventional therapies (surgical resection with or without chemotherapy) [1]. Nonetheless, approximately 30% of RCC patients have metastases at diagnosis [2] and fail to respond to cytotoxic chemotherapy and targeted chemotherapy [1, 3, 4], resulting in the 5-year survival rate less than 10% [2]. Most of ccRCC is initiated by mutation or missing of *VHL* tumor suppression gene. Function loss of VHL leads to VHL–HIF–mTOR pathway activation [5]. Tyrosine kinase inhibitors targeting VEGF (such as sunitinib and pazopanib) and mTOR inhibitors (such as everolimus and temsiromus) are the standard-of-care therapy for ccRCC patients [6]. However, many patients have progression disease treated with tyrosine kinase inhibitors or mTOR inhibitors. Immune checkpoint inhibitors (such as, nivolumab and ipimumab) have been shown to have acceptable safety and durable antitumor activity in ccRCC clinical treatment [6, 7]. However, only ~ 20% patients had clinical benefits from immune clinical therapy [6, 7]. There is increasing interest investigating non-toxic natural products for various types cancer treatment, searching for natural products with fewer side effects for developing adjunctive therapeutic options is urgently necessary [8].

6-Gingerol, 1-[4′-hydroxy-3′-methoxyphenyl]-5-hydroxy-3-decanone, is a major pharmacologically active ingredient of ginger [9, 10]. Compared to 6-shogaol, 8-gingerol, and 10-gingerol (three other phytochemicals in ginger), 6-gingerol is reported to exert a wide array of biochemical and pharmacological actions, including antibacterial, anti-inflammatory, antioxidant, and antitumor capabilities [11–16]. Evidence has shown, for example, that 6-gingerol can induce cell-cycle G2-phase arrest and apoptosis by activating caspases 3 and 7 in oral and cervical tumor cells [17], stimulate autophagy via drug–DNA interaction and caspase-3-mediated apoptosis in HeLa cells [16], inhibit cell proliferation though mitogen-activated protein kinase (MAPK)-activator protein 1 (AP-1) signaling in colon cancer [13], and suppress metastasis in breast cancer [18]. Despite its activity against oral and cervical cancer, colorectal cancer, and breast cancer, the molecular mechanism and in vivo antitumor properties are still sketchy, and there are no reports about 6-gingerol’s antitumor effects in RCC.

In this study, we focused on the mechanism of 6-gingerol action on RCC in vitro and its antitumor effect in vivo. We found that 6-gingerol can inhibit cell growth by stalling the cell cycle at the G1–S transition via the AKT–GSK 3β–cyclin D1 pathway in vitro. Moreover, 6-gingerol can serve as a single agent for killing RCC cells in vitro and in vivo. Thus, our study suggests that 6-gingerol may be a promising agent for the treatment of RCC.

## Materials and methods

### Cell culture

786-O, 769-P, and ACHN cells were purchased from American Type Culture Collection (Manassas, VA) and maintained in RPMI 1640 (Gibco) containing 10% (v/v) of fetal bovine serum (Hyclone) in a humidified incubator at 37 °C and 5% CO_2_.

### Chemicals

6-Gingerol (Selleckchem) was dissolved in dimethyl sulfoxide (DMSO) or corn oil. Phalloidin (Abcam) was dissolved in DMSO.

### MTT assay

786-O, 769-P, and ACHN cells (at 4000/well) were seeded in 96-well plates. After 24 h, 6-gingerol was added to the medium to achieve the indicated concentrations (0, 10, 20, 30, 40, and 50 μM) in triplicate for 24, 48, and 72 h incubation with the three cell lines. Subsequently, 20 μL of MTT (5 mg/mL in phosphate-buffered normal saline; PBS) was added into each well, and the cells were incubated for 4 h. Then, cell formazan and the growth inhibitory rate were quantified and calculated as described before [19].

### Colony formation assay

786-O, 769-P, and ACHN cells were harvested, counted, and seeded in 6-well plates at 1000/well, and then, the cells were treated with 6-gingerol and maintained in the humidified incubator for 7 days until visible colonies appeared. After that, a 0.1% v/w crystal violet solution (Sigma) was used to stain the cells, and the colony formation capacity of the cells was tested in two-dimensional (2D) culture.

### Cell-cycle assay

Human RCC cell lines 786-O, 769-P, and ACHN were grown in 6 cm dishes. The next day, the cells were treated with 6-gingerol at the concentrations indicated in the figure legends for 48 h. As described above, the cells were harvested, washed, and fixed overnight in 70% ethanol at 20 °C. Then, the cells were washed with PBS, incubated with propidium iodide (30 mg/mL) for 30 min, and were analyzed by flow cytometry on a FACS Calibur flow cytometer (BD Bioscience). The data were analyzed in the Cell Fit software.

### Immunofluorescence assay

786-O, 769-P, and ACHN cells were harvested, counted, and seeded in an 8-well chamber slide. After 24 h, the cells were treated with 6-gingerol at the concentrations indicated in Fig. 3 legends for 48 h, and a Ki-67 protein indirect immunofluorescence assay was performed as described before [20]. Briefly, the cells were incubated with an anti-Ki-67 antibody (Proteintech, #27309-1-AP) overnight at 4 °C (primary antibody), washed with PBS, and then incubated with a secondary antibody (a fluorescein isothiocyanate-conjugated affinity-purified goat anti-rabbit IgG antibody, cat. #ZF-0315, Beijing Zhongshan Golden Bridge Biotechnology) for 60 min at room temperature, followed by F-actin and DAPI staining (1:5000) for 15 min in the dark. The cells were examined by laser scanning confocal microscopy (Nikon A1R/A1).

### Western blotting analysis

786-O, 769-P, and ACHN cells were harvested, and total cellular protein was extracted with RIPA (radioimmunoprecipitation assay) buffer [50 mM Tris (pH 8.0), 150 mM NaCl, 0.1% of SDS, 1% of NP-40, and 0.5% of sodium deoxycholate] containing protease inhibitors: 1% of the Protease Inhibitor Cocktail and 1 mM PMSF (phenylmethanesulfonyl fluoride) (Sigma). As previously described [19], 30 μg of total protein was separated by sodium dodecyl sulfate polyacrylamide gel electrophoresis (SDS-PAGE) on a 12% gel and blotted onto nitrocellulose membranes. The membranes were blocked with 5% skim milk in PBS at room temperature for 1 h and then incubated at 4 °C overnight with a primary antibody, including an anti-Ki 67 (dilution, 1:1000, #19972-1-AP), anti-AKT antibody (dilution, 1:1000; #4685), anti-phospho-Akt Ser473 antibody (dilution, 1:2000; #4060), anti-GSK 3β antibody (dilution, 1:2000; #12456), anti-p-GSK 3β Ser 9 antibody (dilution, 1:2000; #5558), anti-CDK4 antibody (dilution, 1:1000; #12790), and anti-cyclin D1 antibody (dilution, 1:1000; #2926 all from Cell Signaling Technology, Inc., Danvers, MA, USA). Immunoblotting for glyceraldehyde-3-phosphate dehydrogenase (GAPDH, dilution 1:10,000; #KC-5G4; Kang Chen Biotech) was performed as an internal control. Next, the membranes were incubated with a secondary antibody: a horseradish peroxidase-conjugated anti-rabbit IgG antibody (#ZB‐2301; dilution, 1:2000; Beijing Zhongshan Golden Bridge Biotechnology) or a horseradish peroxidase-conjugated anti-mouse IgG antibody (#ZB-2305; dilution, 1:2000; Beijing Zhong-shan Golden Bridge Biotechnology) at room temperature for 1 h. Protein bands were detected with the Western Bright Quantum HRP Substrate Kit (Advansta) and visualized via a Molecular Imager ChemiDoc XRS system (Bio-Rad Laboratories).

### Animal experiments

These experiments were approved by the institutional review board of the First Affiliated Hospital of Xi’an Jiaotong University. Eighteen BALB/c nude mice (4 weeks, male) were randomly separated into three groups, and then, these nude mice were subcutaneously injected with 2 × 10^6^ 786-O cells into the right shoulder. After 3 days, the nude mice were treated with corn oil, 6-gingerol (diluted in corn oil, 2.5 mg/[kg body weight]), or 6-gingerol (diluted in corn oil, 5 mg/[kg body weight]) every 3 days by gavage. Kinetics of tumor formation were estimated by measuring tumor size and volume every 3 days for 38 days along with the body weight of the mice. Tumor volume was calculated using the following equation: tumor volume = length × width × height × 0.523. At the end of the experiment, the animals were euthanized, and tumor tissues were surgically excised from the nude mice. The tumors were weighed and divided into two parts, one part was fixed in 4% paraformaldehyde and embedded in paraffin, whereas from the other part, total protein was extracted as described above by means of a tissue grinder machine (Servicebio, China).

### Pharmacokinetics of 6-gingerol in vivo

These experiments were approved by the institutional review board of the First Affiliated Hospital of Xi’an Jiaotong University. 54 BALB/c nude mice (4 weeks, male) were randomly separated into two groups, and treated with 6-gingerol (2.5 mg/[kg body weight], 5 mg/[kg body weight]) by gavage, or corn oil for control. Pharmacokinetics of 6-gingerol in vivo were measured at 9 timepoints (0, 0.5, 1, 1.5, 3, 4, 6, 12, and 24 h), and each timepoint had 3 mice for measure. Mice were euthanized at every interval of the timepoints, and then blood was collected by cardiac puncture, centrifuged at 3000 rpm for 15 min to obtain plasma, and then stored at − 80 °C before use.

The concentration of 6-gingerol in plasma was detected with Chromatograph (UltiMate 3000 RS, Thermo Fisher Scientific, China) and mass spectrometer (TSQ Quantum Triple Quadrupole Mass Spectrometer, Thermo Fisher Scientific, China) by Servicebio Technology Company (Wuhan, Hu Bei, China). For sample preparation, 30 μL of plasma was extracted with 90 uL methanol, and centrifuged at 12000 rpm for 10 min, and the supernatant was then taken for further analysis. For the concentration of 6-gingerol detection, the mass spectrometry detection conditions are as follows: ion source, electrospray ionization source (ESI); scanning method, positive and negative ion switching scanning; detection method, select reaction monitoring (SRM); electrospray voltage (spary voltage), 4000 V (Positive); capillary temperature, 350 °C; collision gas, high-purity argon (purity ≥ 99.999%); sheath gas, nitrogen (purity ≥ 99.999%), 50 Arb; Aux Gus Pressure, nitrogen (purity ≥ 99.999%), 15 Arb; and data collection time, 5 min. The liquid chromatography detection conditions are as follows: column, thermo hypersil GOLD 100 × 2.1 mm, 1.9 μm; flow rate, 0.5 mL/min; aqueous phase, 10 mM ammonium acetate (containing 0.1% acetic acid); organic phase, methanol; needle washing solution, methanol; column oven temperature, 40 °C; autosampler temperature, 10 °C; syringe height, 2 mm; autosampler cleaning setup, both; autosampler needle volume, 200 μL; immersion time of the autosampler needle cleaning, 3 ms; autosampler injection volume, 10 μL; chromatographic gradient, timepoint (0, 2, 3.5, 3.8, 5 min), water phase (60, 5, 5, 60, 60%), and organic phase (40, 95, 95, 40, 40%).

### Immunohistochemical analysis

Five-micrometer-thick sections of animal tumor tissues were prepared. A DAKO Autostainer Plus system was employed to perform immunohistochemical staining of p-AKT Ser473 (Cell-Signaling Technology, dilution 1:100, #4060), GSK 3β (Cell-Signaling Technology, dilution 1:100, #12456), and cyclin D1 (Cell-Signaling Technology, dilution 1:50, #2978). Scoring of each tissue section was performed in a double-blinded manner. Each section was examined under a microscope in high-power fields of view (× 400).

### Statistical analysis

The results were analyzed in the GraphPad Prism software, and the differences between two groups were compared by two-tailed, unpaired Student’s *t* test. *p* < 0.05 was regarded as the threshold value of statistical significance.

## Results

### 6-Gingerol inhibits the growth of RCC cells

**Fig. 1:**
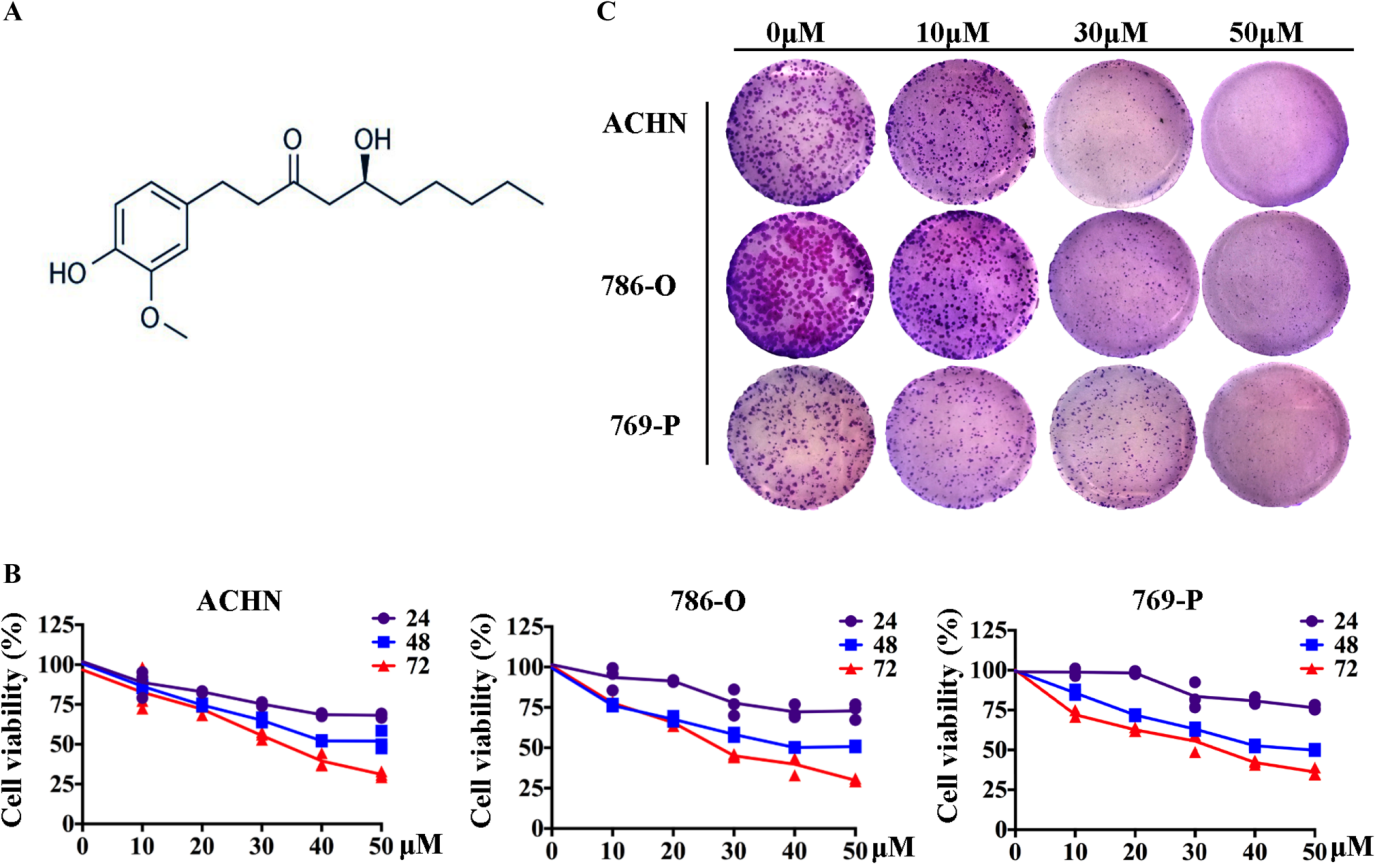
Effects of 6-gingerol on proliferation of RCC cell lines. **a** Chemical structure of 6-gingerol. **b** 6-Gingerol inhibits ACHN, 786-O, and 769-P cell growth in a dose- and time-dependent manner. Cell-viability rates were determined by the MTT assay after RCC cells were treated with the indicated doses of 6-gingerol (0, 10, 20, 30, 40, or 50 μM) for 24, 48, or 72 h. Statistical analysis of the data in triplicate was calculated using student’s *t* test. **c** 6-Gingerol inhibits the colony formation of ACHN, 786-O, and 769-P cells. A thousand cells (per well) were seeded in a 6-well plate and treated with 30 or 50 μM 6-gingerol, and then the cells were stained with crystal violet after 7 days

The chemical structure of 6-gingerol is shown in Fig. 1a. To investigate the influence of 6-gingerol on cell proliferation, ACHN, 786-O, and 769-P RCC cells were treated with 6-gingerol (0, 10, 20, 30, 40, or 50 μM). The MTT assay revealed that 6-gingerol can obviously suppress cell growth in a dose-dependent and time-dependent manner in the three cell lines (Fig. 1b). Half-maximal inhibitory concentration (IC_50_) values of 6-gingerol after 72 h treatment of ACHN cells were found to be 27.41 μM, 31.05 μM for 786-O cells and 30.48 μM for 769-P cells. We chose 10 μM as a low dose of 6-gingerol for cell treatment, 30 μM as IC_50_ for RCC cells, and 50 μM for high-dose treatment with 6-gingerol in all the subsequent experiments. The clonogenic potential of RCC cells was determined by a colony formation assay. As depicted in Fig. 1c, 6-gingerol inhibited colony growth in a dose-dependent manner in ACHN, 786-O, and 769-P cells.

### 6-Gingerol blocks cell cycle by arresting it at the G1 transition

**Fig. 2:**
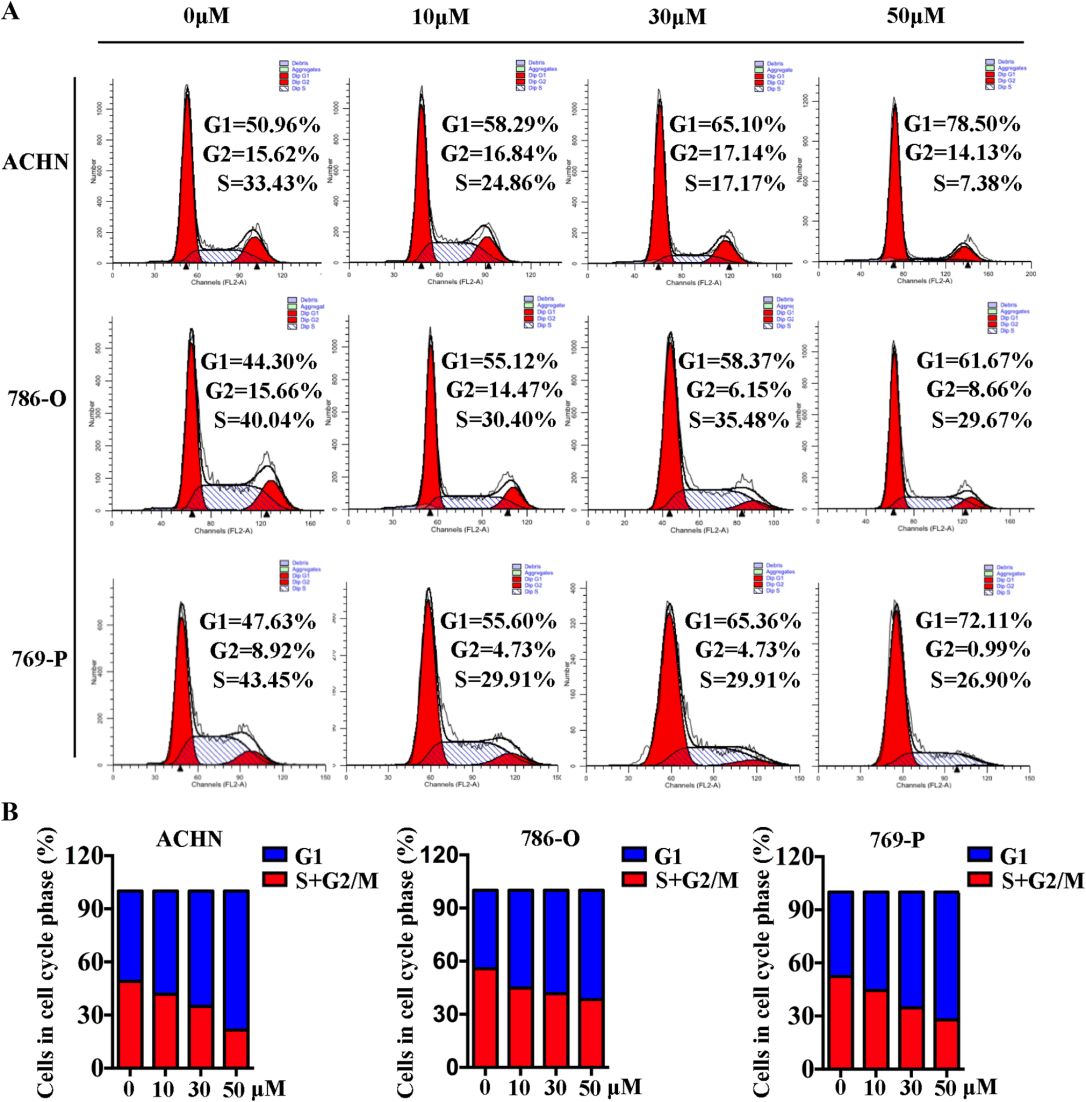
6-Gingerol induces cell cycle arrest in ACHN, 786-O, and 769-P cells. **a** 6-Gingerol caused G1 phase arrest in ACHN, 786-O, and 769-P cells. Cells were seeded in 6 cm dishes and treated with 30 or 50 μM 6-gingerol for 48 h. DNA content was evaluated by propidium iodide (PI) staining and flow cytometry. **b** Percentages of cells in G1 and S/G2 + M phases of the cell cycle are shown in the bar diagram

Next, to determine the mechanism of 6-gingerol action on renal cancer cell growth, DNA-based cell-cycle analysis was performed on ACHN, 786-O, and 769-P cells after treatment with 10, 30, or 50 μM 6-gingerol for 48 h. As shown in Fig. 2a, b, the indicated concentrations, 0, 10, 30, and 50 μM 6-gingerol, resulted in enhanced accumulation of cells at the G1 transition, i.e., 50.96% 58.29%, 65.10%, and 78.50% of ACHN G1 phase cells; 44.30%, 55.12%, 58.37%, and 61.67% of 786-O G1 phase cells; and 47.63%, 55.60%, 65.36%, and 72.11% of 769-P G1 phase cells, respectively.

### 6-Gingerol reduces Ki-67 nuclear staining in RCC cells

**Fig. 3:**
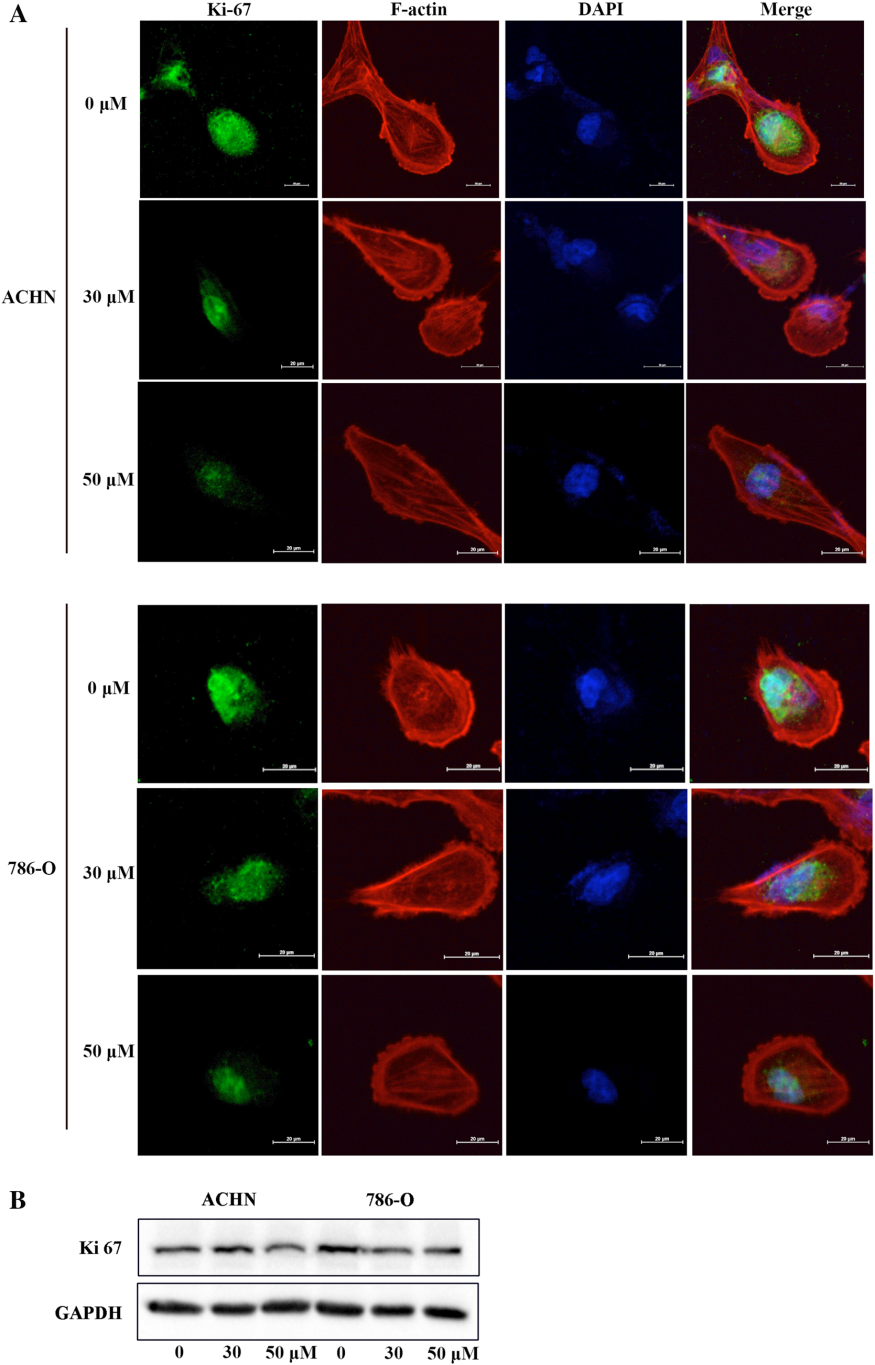
Ki-67 is under expressed after treatment with 6-gingerol. **a** Cells were seeded in an 8-well chamber slide and treated with 0, 30, and 50 μM of 6-gingerol for 48 h. Then, Ki-67 immunofluorescent staining (green), F-actin staining (red) for the cytoskeleton, and DAPI (blue) staining for cell nuclei were all photographed by a laser confocal microscope. **b** ACHN and 786-O cells were seeded in 10 cm dishes and treated with 0, 30, and 50 μM of 6-gingerol for 48 h. Then, the cells were lysed, and the protein levels of Ki-67 were analyzed by a western blot assay

Ki-67 is known to be a cell proliferation marker for research and cancer histopathology and a marker for a cell response to drugs that target cell proliferation [21, 22]. In our study, Ki-67 immunofluorescent staining was evaluated to reveal the RCC cell response to 6-gingerol. As expected, Ki-67 immunofluorescent staining was weakened in a dose-dependent manner in ACHN and 786-O cells after treatment with 30 μM or 50 μM 6-gingerol for 48 h (Fig. 3).

### 6-Gingerol reduces AKT phosphorylation and cyclin D1 and CDK4 expression in RCC cells

**Fig. 4:**
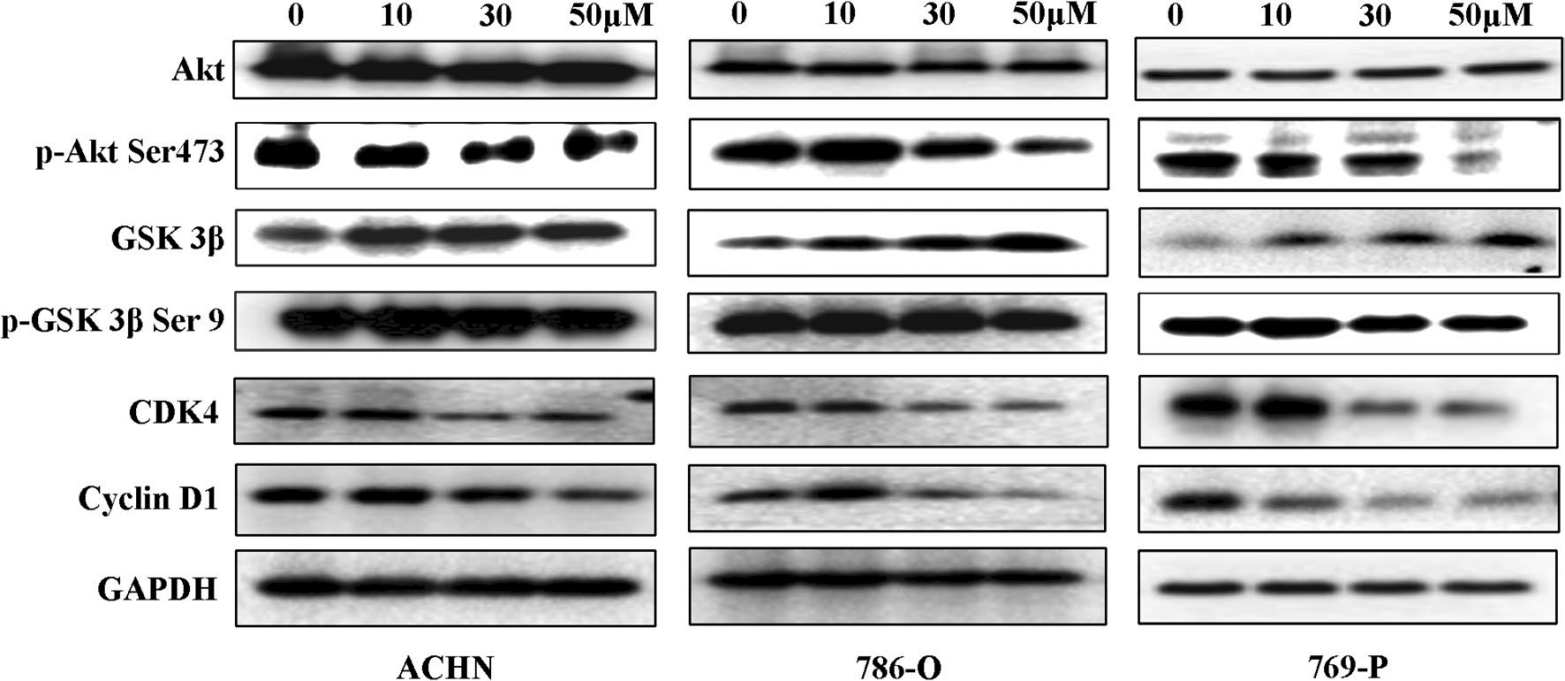
AKT–GSK 3β–cyclin D1-signaling pathway is involved in 6-gingerol-induced G1 arrest of ACHN, 786-O, and 769-P cells. Cells were seeded in 10 cm dishes and treated with 30 or 50 μM 6-gingerol for 48 h. Then, the cells were lysed, and the protein levels of AKT, p-AKT Ser 473, GSK 3β, p-GSK 3β Ser 9, cyclin D1, and CDK4 were analyzed by a western blot assay

The expression of cyclin D1 and CDK4, which forming a complex with CDK6 for the G1–S phase transition [23], was measured by western blotting. As illustrated in Fig. 4, cyclin D1 and CDK4 protein levels were markedly downregulated in ACHN, 786-O, and 769-P cells after treatment with 30 or 50 μM 6-gingerol for 48 h. Western blotting also revealed that the signaling proteins upstream of cyclin D1, and p-AKT Ser 473 was inhibited and GSK 3β was upregulated by 30 or 50 μM 6-gingerol treatment. The above results suggested that 6-gingerol could suppressed AKT–GSK 3β signaling and, furthermore, reduced the expression of cyclin D1 and caused G1 arrest in RCC cells.

### 6-Gingerol inhibits tumor xenograft growth through AKT–GSK 3β signaling

**Fig. 5:**
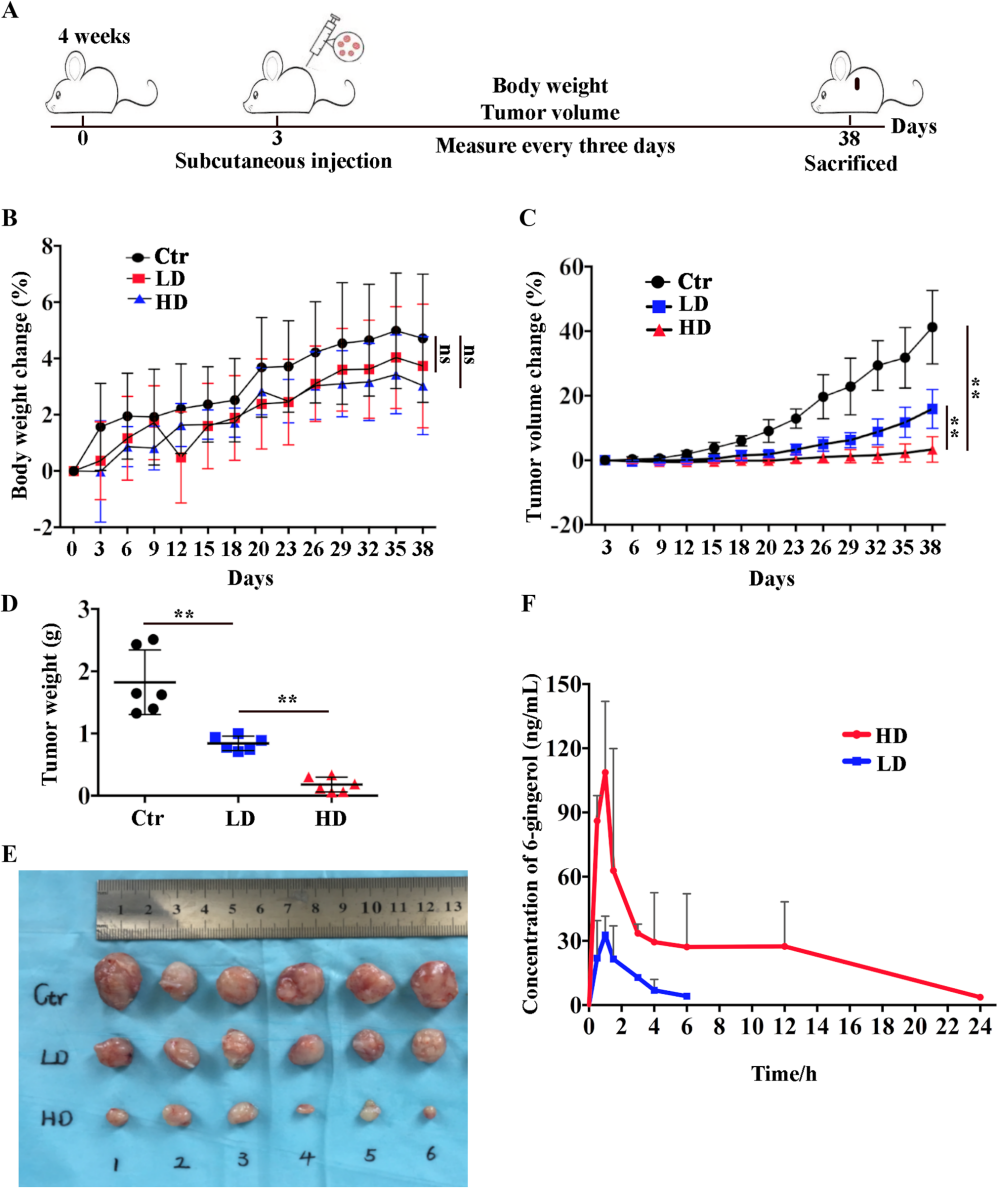
6-Gingerol inhibits tumor growth in vivo. **a** Protocol for tumor-bearing BALB/c nude mice treated with 6-gingerol. 18 BALB/c nude mice were randomly separated into three groups and treated with vehicle (corn oil; control, *n* = 6), 2.5 mg/kg 6-gingerol (*n* = 6), or 5 mg/kg 6-gingerol (*n* = 6) every 3 days after subcutaneous injection of tumor cells until euthanasia. **b** Body weight changes of the xenografted mice treated or not treated with 6-gingerol; Statistical analysis of the data was calculated using student’s *t* test between two groups, error bars indicate ± SD, *n* = 6. *ns* no significance. **c** Tumor volume changes in the xenografted mice during the treatment. Statistical analysis of the data was calculated using student’s *t* test between two groups, error bars indicate ± SD, *n* = 6. **p < 0.01 as compared with the control group. **d** Tumor weight of xenografted mice treated or not treated with 6-gingerol. Statistical analysis of the data was calculated using student’s *t* test between two groups, error bars indicate ± SD, *n* = 6. **p < 0.01 as compared with the control group. **e** Tumor images from groups treated or not treated with 6-gingerol. Ctr represents control group, *n* = 6; LD represents 2.5 mg/kg 6-gingerol treatment group, *n* = 6; and HD represents 5 mg/kg 6-gingerol treatment group, *n* = 6. **f** Plasma concentration–time curves of 6-gingerol. 6-gingerol was given by gavage, and blood was obtained and detected as described in the “Methods”. LD represents 2.5 mg/kg 6-gingerol treatment group (*n* = 3), and HD represents 5 mg/kg 6-gingerol treatment group (*n* = 3). Statistical analysis of the data was calculated using student’s *t* test between two groups, error bars indicate ± SD

The antitumor effect of 6-gingerol in vivo was validated in the 786-O xenograft model. The nude mice were subcutaneously injected with 2 × 10^6^ cultured 786-O cells, and the tumor-bearing mice were treated with corn oil, a low dose of 6-gingerol (2.5 mg/kg), or a high dose of 6-gingerol (5 mg/kg) every 3 days; meanwhile, the tumor growth was monitored by means of tumor volume and tumor weight (Fig. 5a). We observed that the mice treated with 6-gingerol (2.5 or 5 mg/kg) experienced no significant body weight loss relative to the corn oil group (Fig. 5b). On the contrary, the mice from the 6-gingerol 2.5 and 5 mg/kg groups gained some weight (Fig. 5b). Moreover, tumor growth was inhibited significantly between days 18 and 38 (Fig. 5c). The experiment and treatment were stopped on day 38 when one control mouse had an almost 2 cm diameter tumor on its shoulder, even though no mice showed clear signs of ill health due to 6-gingerol treatment or tumors. As expected, tumor weight and tumor volume were significantly changed at the final timepoint (day 38; Fig. 5d, e). The concentration of 6-gingerol in plasma was also measured in mice, and the main pharmacokinetic parameters of 6-gingerol were shown in Fig. 5f, and Table S1. In high dose of 6-gingerol group, the half-time and Tmax of 6-gingerol were about 3.6 h and 1.5 h, respectively, and *C*_max_ was (181.37 ± 76.31) ng/mL. In low dose of 6-gingerol group, the concentrations of 6-gingerol at 12 h and 24 h were not detectable; thus, the pharmacokinetic parameters could not be calculated.

**Fig. 6:**
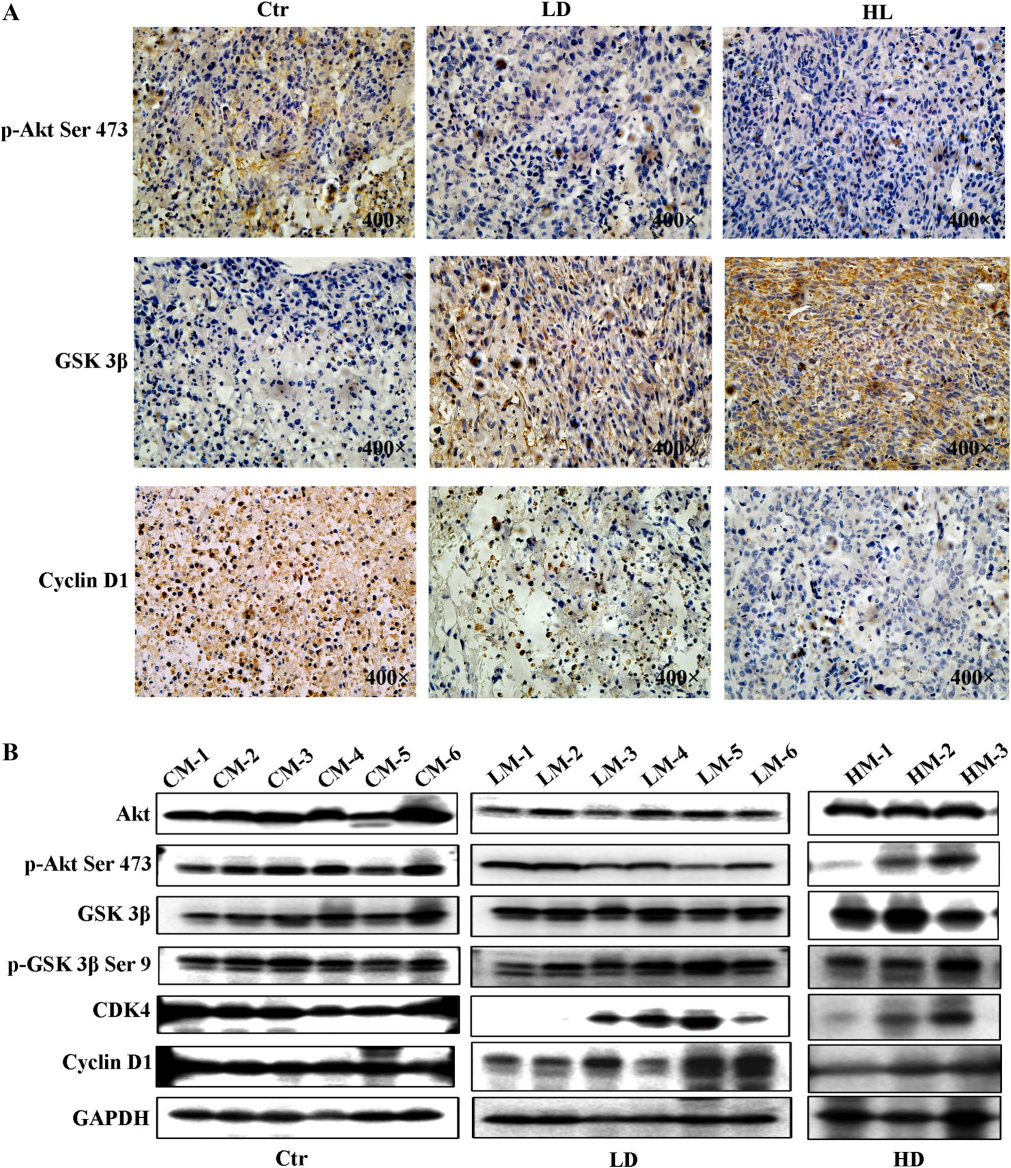
6-Gingerol suppresses the AKT–GSK 3β–cyclin D1-signaling pathway in vivo. **a** Immunohistochemical staining of p-AKT Ser 473, GSK 3β, and cyclin D1 in subcutaneous tumors with or without 6-gingerol treatment. **b** AKT, p-AKT Ser 473, GSK 3β, p-GSK 3β Ser 9, cyclin D1, and CDK4 expression levels were detected by a western blot assay in subcutaneous tumors from the mice with or without 6-gingerol treatment. Ctr represents control group (*n* = 6), LD represents 2.5 mg/kg 6-gingerol treatment group (*n* = 6), and HD represents 5 mg/kg 6-gingerol treatment group (*n* = 6). CM-1 means control group mouse and the mouse numbered 1, LM-1 means 2.5 mg/kg 6-gingerol treatment group mouse and the mouse numbered 1, HM-1 means 5 mg/kg 6-gingerol treatment group mouse and the mouse numbered 1

The expression levels of AKT, p-AKT Ser 473, GSK 3β, p-GSK 3β Ser 9, CDK4, and cyclin D1 in tumor tissue removed from the mouse groups treated or not treated with 6-gingerol were measured next. As presented in Fig. 6a, both doses of 6-gingerol attenuated immunohistochemical staining of p-AKT Ser 473 and cyclin D1 in the tumor tissues, whereas strong staining of GSK 3β was observed in high dose of 6-gingerol treatment group. We also measured AKT, p-AKT Ser 473, GSK 3β, p-GSK 3β Ser 9, CDK4, and cyclin D1 in tumor tissue by western blotting (Fig. 6b). The above results suggested that 6-gingerol can inhibit tumor cell growth by downregulating cyclin D1 through inhibition of the Akt–GSK 3β-signaling pathway in vivo.

## Discussion

Our study addressed the anticancer capacity of 6-gingerol against RCC in vitro and in vivo. The results proved that 6-gingerol inhibits RCC growth (Fig. 1). Furthermore, we found that 6-gingerol suppresses cell growth primarily by inhibiting the AKT–GSK 3β–cyclin D1 pathway, as evidenced by a significant decrease in p-AKT Ser 473, CDK4, and cyclin D1 protein amounts, meanwhile, a significant increase GSK 3β protein amounts in vitro and in vivo (Figs. 4, 5, 6), with concentration-dependent accumulation of cells at the G1 phase in vitro (Fig. 2). The above results are consistent with those of other studies about 6-gingerol effects on colon cancer cells, in which 6-gingerol induced the G2–M arrest of the cell cycle at concentrations (34–51 μM) similar to those in our study [13].

Some studies have proved that 6-gingerol inhibits the growth of other cancer cells at concentrations as high as 500 μM in the culture medium [17]. Note that 125 μg/mL (424 μM) 6-gingerol is IC_50_ reported for HeLa cells [16]; those authors reported that 6-gingerol induces apoptosis and autophagy by binding to DNA [16]. Moreover, other investigators have studied a high concentration (up to 200 μM) of 6-gingerol as a therapeutic agent killing HT-29, HCT-116, SW480, and Caco-2 cells [24]. Nevertheless, 5–15 μg/mL (up to 50 μM) 6-gingerol has been used to treat LoVo cells [25], and the results showed that a low concentration of 6-gingerol (34 μM) can cause significant growth inhibition in agreement with our results (Fig. 2). The possible reasons for the discrepancies between these studies and our study may be the differences in the purity of 6-gingerol provided by different companies, differences in sensitivity of different cell lines, differences in 6-gingerol duration of treatment of different cell lines, and dissimilarity of the mechanisms of action in different cell lines.

6-Gingerol has been previously shown to inhibit tumor cell growth through downregulation of cell-cycle regulators CDK1, cyclin A, and cyclin B1 and by repressing cyclin D1 in colorectal cancer cells [24], by activating caspases 3 and 9 and modulating mitochondrial functions in human gastric cancer cells [11], by inhibiting protein expression of the cyclin family and mTOR pathway in human cervical adenocarcinoma cells [15], by decreasing cyclin A and CDK expression, and by altering MAPK and PI3K–AKT pathways in pancreatic cancer cells [12]. The previous research results have indicated that the AKT–GSK 3β pathway and cyclins may be the common mechanism of action of 6-gingerol in its antitumor activities. Nevertheless, there is no study about the antitumor action of 6-gingerol in RCC.

In our experiments, we also found that the AKT–GSK 3β–cyclin D1-signaling pathway is involved in the cell-cycle arrest induced by 6-gingerol. Because cyclin D1 is the key cell-cycle regulatory protein controlling the G1 cell-cycle phase to the S phase [26, 27], we measured its expression after a G1 cell-cycle arrest was observed in ACHN, 786-O, and 769-P cells (Fig. 2a). As expected, cyclin D1 protein expression was decreased in a dose-dependent manner by 6-gingerol treatment in our study (Fig. 4), and these results were consistent with the reports by Seong-Ho Lee et al. about human colorectal cancer cells [24]. Furthermore, evidence indicated that GSK 3β controls the cell cycle by regulating cyclin D1 degradation [28, 29], whereas GSK 3β is one of the major effectors of AKT and is inactivated by phosphorylation at Ser9 by AKT [30]. In our study, proteins p-AKT Ser 473 and cyclin D1 were significantly downregulated after 50 μM 6-gingerol treatment of RCC cells (Fig. 4). Meanwhile, protein of GSK 3β was increased after 50 μM 6-gingerol treatment (Fig. 4). The decreased protein amounts of p-AKT Ser 473 and cyclin D1 and increased protein amount of GSK 3β were detected by western blotting and immunohistochemical staining in the tumor tissue removed from the mice treated with 6-gingerol as compared to that removed from control untreated mice (Fig. 6). Therefore, we concluded that 6-gingerol induces the G1 cell cycle arrest of RCC cells though interaction with the AKT–GSK 3β–cyclin D1-signaling pathway in vivo and in vitro.

As far as we know, ginger is a food product and has been used in medicine [31]. In our study, a significant decrease in tumor weight and tumor volume was observed in tumor-bearing mice treated with 6-gingerol at 2.5 and 5 mg/kg doses (Fig. 5). Nonetheless, our results suggest that the higher dose has no effect on mouse body weight and lifespan when we compared the control group and the 6-gingerol treatment group (Fig. 5b). These findings were verified in other cancers. Because there was no detectable cytotoxicity of 6-gingerol in vivo, the anticancer capacity of 6-gingerol should be evaluated in future animal studies and clinical trials.

## Conclusion

Our results indicate that 6-gingerol, as a natural compound, exerts an antitumor action by inhibiting the cell cycle through the AKT–GSK 3β–cyclin D1-signaling pathway in RCC. Various data combined with our study suggest that 6-gingerol may be a safe and useful complementary therapy for ccRCC and warrant further investigation of its antitumor efficacy in vivo and in vitro, either alone or in combination with TK inhibitors and mTOR inhibitors.

## Electronic supplementary material

Below is the link to the electronic supplementary material.

Supplementary material 1 (PDF 117 kb)
