# Supplementary material for: 6-Gingerol induces cell-cycle G1-phase arrest through AKT–GSK 3β–cyclin D1 pathway in renal-cell carcinoma
Source: Cancer Chemother Pharmacol. 2019 Dec 12;85(2):379–90. doi: 10.1007/s00280-019-03999-9 (PMC7015962; doi:10.1007/s00280-019-03999-9)
Supplement: Supplementary file 1 — Supplementary material 1 (PDF 117 kb) [file 280_2019_3999_MOESM1_ESM.pdf]

Table S1 Pharmacokinetic parameters of 6-Gingerol (n=3)

	5 mg/kg		2.5 mg/kg	
Parameters	Values	SD	Values	SD
Tmax/h	1.5	1.32	4.38	2.31
Cmax/(ng/mL)	181.37	76.31	33.88	15.97
AUC/(h*ng/mL)	698.74	461.02	36.04	17.39
AUC (0-∞)/(h*ng/mL)	723.14	463.27	76.10	NA
λz/(1/h)	0.2	0.05	0.39	NA
t1/2/h	3.64	1.07	1.76	NA
Vz/(mL/kg)	50366.36	37104.63	83384.74	NA
Cl/(mL/h/kg)	10497.96	8851.56	32849.92	NA
MRT/h	5.41	3.69	3.76	3.81
